# A Modified Method for Transient Transformation via Pollen Magnetofection in *Lilium* Germplasm

**DOI:** 10.3390/ijms242015304

**Published:** 2023-10-18

**Authors:** Mingfang Zhang, Xu Ma, Ge Jin, Dongyang Han, Jing Xue, Yunpeng Du, Xuqing Chen, Fengping Yang, Chunli Zhao, Xiuhai Zhang

**Affiliations:** 1 Beijing Academy of Agriculture and Forestry Sciences, Key Laboratory of Urban Agriculture (North China), Ministry of Agriculture and Rural Affairs, Beijing 100097, Chinachenxuqing@baafs.net.cn (X.C.); zhangxiuhai@baafs.net.cn (X.Z.); 2 College of Forestry and Grassland Science, Jilin Agricultural University, Changchun 130118, China

**Keywords:** *Lilium regale*, genetic transformation system, pollen magnetofection, PBI121 and pYBA1132 transformation

## Abstract

Lily (*Lilium* spp.) is a popular ornamental plant. Traditional genetic transformation methods have low efficiency in lily, thus development of a high-efficiency genetic transformation system is important. In this study, a novel transient transformation method involving pollen magnetofection was established and optimized pollen viability, and exogenous gene expression in magnetofected pollen and that of different germplasm were assessed. The highest germination percentage of *Lilium regale* pollen was 85.73% in medium containing 100 g/L sucrose, 61.5 mg/L H_3_BO_3_, and 91.5 mg/L CaCl_2_. A 1:4 ratio of nanomagnetic beads to DNA plasmid and transformation time of 0.5 h realized the highest transformation efficiency (88.32%). The GFP activity in transformed pollen averaged 69.66%, while that of the control pollen was 0.00%. In contrast to the control, transgenic seedlings obtained by pollination with magnetofected pollen showed strong positive GUS activity with 56.34% transformation efficiency. Among the lily germplasm tested, ‘Sweet Surrender’ and *L. leucanthum* had the highest transformation efficiency (85.80% and 54.47%), whereas *L. davidii* var. *willmottiae* was not successfully transformed. Transformation efficiency was positively correlated with pollen equatorial diameter and negatively correlated with polar axis/equatorial diameter ratio. The results suggest that pollen magnetofection-mediated transformation can be applied in *Lilium* but might have species or cultivar specificity.

## 1. Introduction

Plant genetic transformation is an important pathway to improve plant yield, quality, and tolerance to abiotic/biotic stress [1]. Plant transformation is a complex process that involves the preparation of explants, delivery of genes of interest into plant cells via *Agrobacterium*- or biolistic-mediated methods, selection and regeneration of transgenic plants, and verification of gene integration into the genome through molecular methods. Numerous factors are known to affect the efficiency of plant transformation and these have been well summarized in previous reviews [2,3,4]. The main bottleneck in successful plant genetic transformation is how biomolecules enter plant cells through the hard multi-layer cell wall and the subsequent regeneration of transgenic plants from an in vitro-cultured explant, either via de novo organogenesis or somatic embryogenesis [5]. Genetic transformation remains challenging in many plant species; the recalcitrance of cells to in vitro culture and their propensity to divide and regenerate on synthetic media represent the most serious obstacle to the development of effective plant transformation systems [6]. Different species respond disparately to tissue culture conditions. Among the challenges associated with plant transformation, low rates of transformation and genotype dependence are two major factors that cause plant transformation to be a bottleneck for transgenic approaches.

Since the first transgenic plants were reported in 1983, research on plant genetic transformation has burgeoned [7]. Genetic transformation is an important technical means to study gene function. In 1992, Cohen and Meredith first transformed lily bulbs using the *Agrobacterium*-mediated method and detected foreign genes in tumor-like protrusions [8]. Agrobacterium-mediated transformation provides several benefits, such as preferential integration into transcriptionally active regions of the chromosome, a potentially low copy number, and the defined integration of transgenes, compared with the direct gene delivery methods [9]. However, owing to strong genotype dependence, poor genetic stability, and the difficulty of regenerating and propagating trans-genetic explants, this technology cannot meet the requirements of genetic engineering for gene function verification and trait improvement.

Particle bombardment (also known as a gene gun) is a physical method of introducing exogenous DNA directly into the plant genome [10]. With many innovations, gene-gun-mediated, together with *Agrobacterium*-mediated, gene transformation methods remain the most commonly used. It is not limited by the source of receptor materials; cells, calli, immature embryos, and organs can all be used as targets for transformation. However, the particle-bombardment-mediated gene transformation method can only transfer DNA fragments smaller than 10 kb because larger fragments are easy to break during bombardment or have weak adherence to metal particles, resulting in chaotic DNA integration events [11] and plant tissue damage, rendering regeneration inefficient [12]. It also has the disadvantage of higher costs and unprotected exogenous DNA, which cannot be applied in large-scale genetic engineering.

Apart from the two major methods detailed above, exogenous genes can be delivered to plant cells using electroporation and other delivery methods [13,14]. As for the electroporation-mediated gene transformation method, it has the advantages of rapid application, low cost, and a highly stable transformation rate [15]. In addition, unlike particle bombardment, electroporation produces primarily single-copy plasmid fragments [16]. The main disadvantage of electroporation is the difficulty in transforming plant cells with thick cell walls [17], and it only works with a limited number of receptor species. Furthermore, strong electric field pulses can destroy the naked gene, resulting in inaccurate translation of the final product [1].

However, the use of these gene delivery technologies requires establishing in vitro cultures and regeneration protocols. Genetic transformation remains challenging in many plant species; the recalcitrance of cells to in vitro culture and their propensity to divide and regenerate on synthetic media represent the most serious obstacle to the development of effective plant transformation systems [6]. Different species respond differently to tissue culture conditions. Given these challenges, such as low rates of transformation and genotype dependence, and the laborious and time-consuming trial-and-error approaches still prevailing in the development of plant cell and tissue culture protocols, the formulation of transformation methods that obviate the need for tissue culture and regeneration altogether is highly desirable.

Magnetic nanoparticles (MNPs) have become a major research focus and have been widely used in the biomedical field in recent decades owing to their unique magnetic properties [18]. Pollen magnetofection is a recently developed method for establishing a genetic transformation system using pollen grains as the recipient material and is reported to be an effective means of obtaining stable, genetically modified progeny in many species with sufficiently large pollen apertures, including the difficult-to-transform crop plants chili pepper and pumpkin. Zhao et al. first reported successful application of the method in lily, as an example of a monocot species, through staining for β-glucuronidase (GUS) [19]. However, the results of Zuzana et al. [20] indicated that GUS reporters are not ideal because of positive GUS staining in nontransformed pollen grains. These authors used green fluorescent protein (GFP)-based reporter plasmids as an alternative but obtained no indication of plasmid-induced transient expression in maize, sorghum, and lily. Compared to traditional gene delivery methods, nanoparticle-mediated delivery has the advantages of directly crossing bio-membranes, protecting and releasing multiple cargoes, and achieving multidimensional targeting through chemical and physical tunability [21]. Also, this method does not involve protoplast manipulation, cell culture, or plant regeneration processes, and this method mediating DNA transfer is relatively simple, avoiding cell culture and plant regeneration processes inherent in other genetic transformation systems. In addition, this method frequently avoids the drawbacks of poor regenerative ability, genotype limitation, and genetic variation such as mutation and methylation.

Lily is an important cut flower in the floriculture industry. It plays a crucial role in the flower market economy. Genetic engineering has become one of the most applicable strategies for breeding of new lily cultivars through the introduction of desired traits, especially biotic and abiotic stress resistances which affect lily quality, yield, and productivity. Because genetic transformation not only produces an “additive” one-point improvement, but also can modify target traits by direct incorporation of related genes, genetic transformation methods will be used in the near future as standard breeding tools in combination with traditional breeding methods.

It is known that genetic modification of lily is restrained; because of its large genome and high heterozygosity, the gene transformation has always been a big obstacle. Therefore, it is very important to establish an efficient gene delivery system, not only to obtain a better understanding of the key gene functions controlling important traits, but also to accelerate molecular breeding in lily through gene modifications. Furthermore, traditional gene transformation methods such as electroporation bombardment and sonication, *Agrobacterium* infection, or pollen-tube-mediated transformation are genotype-dependent and display low efficiency in lily species [22,23,24,25,26,27]. To our knowledge, the application of pollen transfection in various types of lily germplasm using MNPs has not been reported. In this study, we established a lily pollen transfection system applicable to large-scale, fast, and efficient transfection.

## 2. Results

### 2.1. Pollen Viability

Pollen viability is a crucial factor that significantly affects transformation efficiency. In this study, the PGM was modified to achieve improved germination efficiency: the medium (1 L) contained 100 g of sucrose, 61.5 mg of H_3_BO_3_, and 91.5 mg of CaCl_2_. Germination of fresh *L. regale* pollen in vitro and the growth of pollen tubes were observed. The pollen viability was assessed from the percentage of germination (germination was determined based on the presence of a pollen tube). The highest germination percentage of the collected pollen was 85.73% and the lowest germination percentage was 40.33%, differing significantly (Table 1).

### 2.2. Establishment of Gene Transformation System Using Magnetofected Pollen

#### 2.2.1. Effect of Ratio of Magnetically Driven Plasmids to Plasmid DNA and Transformation Time

To assess the binding ability of exogenous DNA to nanomagnetic beads, different magnetic bead:plasmid mass ratios (1:4, 1:1, and 3:2) and different transformation times (0.5, 1.0, or 1.5 h) were applied. The optimal conditions of the nanomagnetic beads system for transformation of *L. regale* pollen were determined. A nanomagnetic bead:plasmid ratio of 1:4 and transformation time of 0.5 h resulted in the highest transformation efficiency, and the percentage pollen staining for GUS activity after transformation was the highest at 85.50% (Table 2, Figure S3).

#### 2.2.2. Effect of Magnetofected Pollen on Pollen Germination Percentage

The nanomagnetic beads transformation method comprised mild conditions and had little effect on the pollen viability, thus the transformed pollen should be suitable for cross-pollination. The transformed pollen was germinated in vitro and a pollen grain was regarded as germinated if its tube’s length exceeded the diameter of the pollen grain. Owing to the poor pollination utility of wet pollen, it was necessary to collect and dry the transformed pollen before cross-pollination. A 500-mesh nylon cloth was used to filter and dry the pollen. The pollen viability before and after drying was assessed under the same germination conditions as used previously. The average percentage of pollen germination in three microscopic fields was calculated for the transformed pollen and the nontransformed pollen of the control. The pollen viability after transformation remained acceptable and did not differ significantly from that of the control group (Table 3, Figure S2).

### 2.3. Exogenous Gene Expression in Magnetofected Pollen

**Figure 1:**
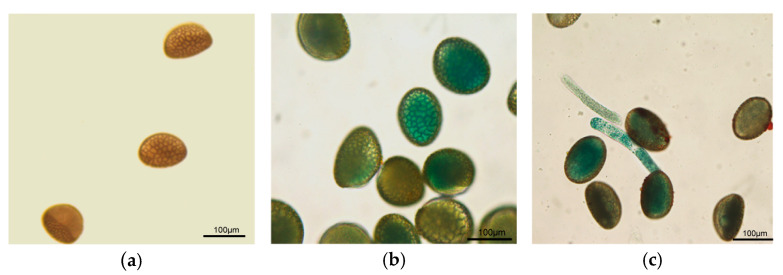
Detection of β-glucuronidase activity in pollen after magnetofection of the PBI121 plasmid. (**a**) Nontreated pollen grains; (**b**) transformed pollen grains; (**c**) germination tube from transformed pollen.

We followed the protocol described by Zhao et al. [19] with a slight adjustment to increase the magnetofection intensity. To test whether exogenous genes can be expressed through magnetofection, we transformed a reporter gene, the pBI121 GUS plasmid, into *L. regale* pollen using MNPs (Figure S1). If the GUS gene is successfully transformed, the GUS protein will be expressed, which can be visualized by staining with X-gluc (5-bromo-4-chloro-3-indolyl-β-D-glucuronic acid) solution. Microscopic observation revealed that lily pollen exhibited GUS activity after transformation with the GUS reporter plasmid. The experiment was independently repeated three times, with similar results (Table 4). Previous experiments with sorghum have shown that positive GUS staining is detected in the control, nontransformed pollen grains [20]. With regard to *L. regale*, a clear difference in GUS activity between the treated and control pollen was observed. Both the pollen cytoplasm and germination tubes showed GUS activity (Figure 1).

**Figure 2:**
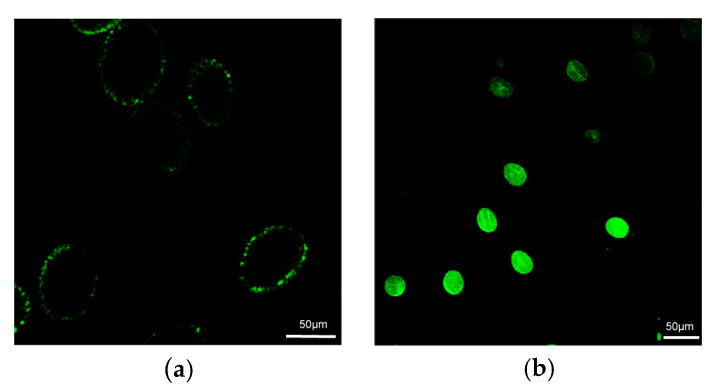
Detection of green fluorescent protein fluorescence in pollen after magnetofection of the pYBA1132 plasmid. (**a**) Nontreated pollen grains; (**b**) transformed pollen grains.

Because staining for GUS is controversial, we detected GFP fluorescence the following day with a laser scanning confocal microscope (Figure S1). We followed a previously published protocol [19], with one modification to use a relatively higher magnetic field intensity. No fluorescence signal was observed in the negative control treatment (i.e., samples transformed with MNPs lacking plasmid DNA; Figure 2). The magnetofection treatment efficiency ranged from 61.32% to 72.56% in the three trials, averaging 69.66% (Table 5). Duncan’s multiple range test, which was used to assess the significance among means in the repeated trials, indicated a statistically significant difference between the negative control and magnetofection with the pYBA1132 plasmid (*p* < 0.05).

### 2.4. Inheritance and Stability in Transgenic Offspring

**Figure 3:**
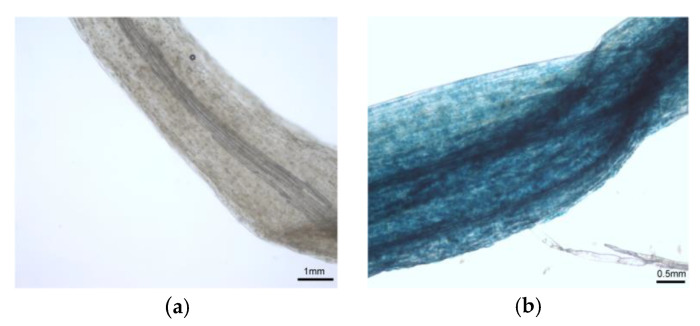
Detection of β-glucuronidase activity in seedling progeny from self-pollination *of L. regale* using magnetofected pollen. (**a**) Control seedling leaf; (**b**) transformed seedling leaf.

**Figure 4:**
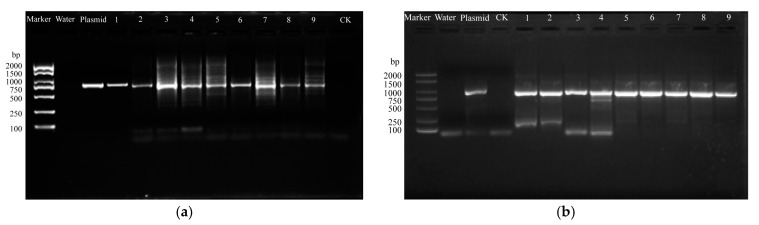
Results of PCR (**a**) and reverse transcription-PCR; (**b**) analyses of the exogenous *GUS* gene in T_1_ transgenic plants raised from self-pollination *of L. regale* using magnetofected pollen. The numbers 1–9 represent different transformed seedlings.

Seeds were obtained from selfing of *L. regale* flowers with magnetofected pollen. After sowing in culture substrate, the seeds germinated and the euphylla developed. GUS activity was detected in the seedling leaves (Figure 3, Figure S5). In contrast to the control, the transformed seedlings showed strong positive GUS activity. The transformation efficiency was 56.34% (Table 6). A molecular analysis of the T_1_ transgenic lily plants was performed, comprising PCR or PCR with reverse transcription (RT-PCR). At the genome level, the PCR results revealed that *GUS* was integrated into the genome of the transgenic plants (Figure 4a). At the transcription level, the RT-PCR results confirmed the successful transcription of *GUS* in the transgenic plants (Figure 4b).

### 2.5. Scanning Electron Microscopic Observation of Pollen Grains from Different Lily Taxa and Hybrids

Because there is evidence that pollen morphology might correlate with transformation efficiency, the morphology of pollen grains from 20 lily taxa and hybrids was observed using scanning electron microscopy (SEM). The following indicators were measured: pollen shape, muri width, sculpture, lumina distribution (LD), polar axis length (P), equatorial diameter (E), P/E ratio (a measure of pollen shape), and P·E (a measure of pollen size). In general, the shape of the pollen grain was oval or elongated oval, and zygomorphic. The lumina shape ranged from an irregular polygon to almost circular. The pollen grain size and shape varied among the materials (Table 7). ‘Bonfire’ pollen had the longest P (133.83 μm), whereas ‘Leichtlinii’ had the shortest P (67.77 μm). ‘Sunderland’ pollen had the longest E (70.43 μm), whereas *L. davidii* var. *willmottiae* had the shortest E (32.60 μm). *Lilium davidii* var. *willmottiae* had the highest P/E value (3.09), while ‘Sunderland’ had the smallest P/E ratio (1.74). ‘Regale’ had the broadest muri (2.40 μm), whereas ‘Cancun’ had the narrowest muri (1.17 μm). ‘Sunderland’ had the largest pollen grains (8643.40 μm^2^), whereas *L. davidii* var. *willmottiae* had the smallest pollen grains (3112.71 μm^2^).

### 2.6. pBI121 Vector Transformation in Magnetofected Pollen of Different Lily Germplasm

Pollen grains from 15 lily taxa and hybrids were successfully transformed by magnetofection using the pBI121 vector (Table 8). Among the lily taxa, *L. leucanthum* had the highest transformation efficiency (54.47%), whereas *L. davidii* var. *willmottiae* was not transformed. Among the lily cultivars, the highest transformation efficiency was for ‘Sweet Surrender’ (85.80%). However, six cultivars were not transformed: ‘Bright Tower’, ‘Pink Flavor’, ‘Bonfire’, ‘Leichtlinii’, ‘Millesimo’, and ‘Fusion’. These results suggested that the pollen magnetofection transformation method might exhibit species or cultivar specificity among members of the *Lilium* genus. In addition, it was noted that five of the lily materials gave false positives during transformation.

Pearson correlation analysis of transformation efficiency with pollen P, E, P/E, and P·E values was performed using the SPSS software v26.0 (Table 9). Transformation efficiency was positively correlated with E and P·E, negatively correlated with P/E, and not significantly correlated with P.

In summary, using the protocol described by Zhao et al., but modified to increase the magnetic field intensity, the feasibility of transient transformation of lily pollen via magnetofection was demonstrated [19]. Although endogenous GUS activity was detected in lily pollen, GUS staining showed a significant difference in activity between the control and magnetofected pollen. We detected strong positive staining for GUS activity in transformed pollen grains and transgenic seedlings obtained through controlled pollination using the transformed pollen. Pollen magnetofection simplifies and shortens the gene transformation cycle for lily, which enables verification of gene functions in lily and facilitates scaled-up production of transformed lily germplasm.

## 3. Discussion

Genetic engineering has played an important role in gene functional analysis and assists breeders to quickly identify target plant traits with a homozygous genetic background, thereby greatly shortening the process of breeding new cultivars. Genetic transformation is certain to join traditional breeding methods as a standard tool for ornamental plant breeding in the near future. To date, plant genetic transformation methods generally used include the *Agrobacterium*-mediated method, pollen tube pathway-mediated transformation, particle bombardment, and virus-induced gene silencing [28,29,30,31,32]. However, *Lilium* spp. is difficult to transform genetically, and only a few transformations have been achieved, which is largely due to a high rate of chimerism and low transformation efficiency [33].

Numerous recent studies have reported the important contributions of these novel technologies for gene transfer to plant tissues and their integration with tissue culture techniques. Nanoparticles comprise nanoscale particles at the atomic or molecular level [34,35]. Nanoparticles behave differently and exhibit different physiochemical properties compared with their bulk counterparts [36]. They are formed from various materials, and their action is dependent on their chemical composition, size, and shape [37]. Nanoparticles not only have been widely applied for bioconjugation with nucleic acids because of their unique electronic, optical, and catalytic properties [38,39,40,41], but also can be used as a vector for gene transfer, including particles based on calcium phosphate, carbon, silica, gold, magnetite, strontium phosphate, magnesium phosphate, and manganese phosphate [42,43,44,45]. Pollen grains are an ideal target for introduction of a foreign gene into the germ line because pollen grains are produced in large numbers and can be easily isolated from the anther and directly transformed by various methods. Furthermore, pollen can not only be used to introduce foreign traits simply by pollination, but also can be used as a natural transformation vector [46,47,48]. Upon contact with stigma, transformed pollen grains of flowering plants hydrate, germinate, and elongate to deliver the sperm cells to the embryo sac for double fertilization [49]. Thus, pollen magnetofection delivers exogenous DNA into the pollen grain and transgenic seeds can be generated by pollination with the transformed pollen.

Pollen viability was one of the key factors that significantly affected the transformation efficiency, and maintaining the maximum pollen viability in the transformation process was the basic condition for the success of the experiment [50]. As for lily pollen, the best germination medium formula varied among different species [51]. The selection of the best in vitro culture medium can make the pollen maintain the best vitality state during magnetic transfection and avoid the loss of germination force due to excessive or too low external osmotic pressure [52]. On the best in vitro culture medium, the pollen germination rate of *Lilium regale* reached 85.73% ± 0.84c. After gene transformation, the viability of the pollen could still remain 60%, which was enough for pollination.

In this experiment, GUS and GFP were both applied as marker genes to evaluate the gene transformation efficiency. The transformation rate of the pollen magnification system is higher compared to previous genetic transformation of DNA directly into the system in lily and the time of the whole process is short. Nishihara et al. transferred the GUS gene into lily using a gene gun, and found the transient expression of GUS using histochemistry [53]. Watad et al. achieved three positive plants after southern blot detection by transferring the PAT gene into the callus using a gene gun [23]. Miyoshi imported the GUS gene into the lily protoplast by electroporation transformation and achieved transient expression but failed to achieve a transformed plant from the protoplast culture [54]. Particle bombardment has been applied for transferring DNA into the scale leaves of *L. davidii* var. *unicolor.* The efficiency of the transformation was determined to be about 1.3% through confirmation using PCR and southern blotting [55]. This method also has some disadvantages, including the requirement of specific equipment and personal skills, a high copy number, and the tendency of DNA sequences to undergo complex rearrangements prior to or during integration [56]. In this research, the transformation efficiency was determined to be 56.34% through confirmation by PCR, which is higher than previous research within a short period. In this experiment, the signals of GUS and GFP can both be detected in both pollen grains and germination tubes (Figure S4), which is consistent with previous research [57].

It has been reported that the genetic transformation of *Lilium* is greatly affected by the genotype of the plant as well as the type and status of the receptor tissue [33]. Among twenty different lily germplasms tested in this experiment, there were six cultivars that could not be successfully transformed. These results suggest that the pollen magnetofection transformation method might exhibit species or cultivar specificity among members of the *Lilium* genus, which is consistent with previous research [8,44,51,58]. Furthermore, according to previous research, it is unclear how DNA alone or associated with nanoparticles is transported into the pollen grains. So, in order to verify the pollen status for optimal magnetofection, it is critical to check the pollen using scanning electron microscopy [59]. In this study, scanning electron microscopy was used to observe the state and morphological structure of pollen of different types of lily germplasm. It was shown that the germination holes were hidden in the germination grooves, not directly exposed to the surface like in cotton and other plants [8]. The results showed that the transformation efficiency was positively correlated with E and P·E, negatively correlated with P/E, and not significantly correlated with P, which indicated that the pollen structure might have an effect on the pollen magnetofection transgenic system. The establishment of this platform will facilitate scaled-up production of transformed germplasm and accelerate molecular breeding through the introduction of new and beneficial traits into *Lilium* species and cultivars.

## 4. Materials and Methods

### 4.1. Materials

In this study, we investigated the effectiveness of transient transformation via pollen magnetofection in lily. One species, *Lilium regale*, was primarily used as the experimental material; this species is an important parent in breeding that is highly valued for its ornamental qualities and exhibits strong stress resistance. Moreover, it is self-fertile and produces readily accessible pollen through protected cultivation, which is suitable for the present approach. PBI121 and a 35S promoter-driven GFP reporter plasmid were selected in this research. To determine the feasibility of the approach in lily, pollen grains from twenty Lilium germplasms, including four species and fifteen cultivars, were collected and treated under the same conditions. Detailed information on the two plasmids with different reporter genes used in the study are provided in the Supplementary Materials and Methods. For plant materials, L. regale was cultivated in our greenhouse. Polyethyleneimine-modified Fe_3_O_4_ MNPs were purchased from Chemicell (PolyMag1000).

### 4.2. Preparation of MNP–DNA Complexes

The MNPs and purified plasmid DNA were diluted to a concentration of 1 μg μL^−1^ with distilled water, then mixed to a mass ratio of 1:4, and further incubated for 30 min at room temperature to form MNP–DNA complexes via electrostatic attraction.

### 4.3. Pollen Magnetofection

The freshly prepared MNP–DNA complexes were dispersed in 1 mL of pollen germination medium to prepare the MNP–DNA complex suspension. The lily pollen germination medium (1 L) contained 100 g of sucrose, 61.5 mg of H_3_BO_3_, and 91.5 mg of CaCl_2_. Lily pollen grains were collected from anthers before dehiscence. Within 12 h, we collected pollen from dehiscent anthers under room temperature. The collected pollen grains were placed in the base of a culture dish and soaked in 1 mL of the MNP–DNA complex suspension; the culture dish was covered and placed on a magnetic stirring apparatus for 0.5 h to magnetofect the pollen grains. The MNP–DNA complexes and pollen suspension were left undisturbed at room temperature. After magnetofection, the suspension was carefully removed using a pipette and the magnetofected pollen was spread onto nylon cloth overlying several layers of filter paper. After drying at room temperature for approximately 30 min, the dried pollen was collected in a tube for pollination.

### 4.4. Expression of an Exogenous Gene in Magnetofected Pollen

To assess whether an exogenous gene can be expressed in lily pollen through application of the magnetofection approach, a P35S::GUS construct was used in this assay. The magnetofected pollen was germinated in PGM at room temperature for 24 h before staining with 0.3% X-gluc solution overnight in the dark, and then washed with 100% ethanol for at least 3 h. The samples were observed with a compound microscope (ZEISS BA 210, Oberkochen, Germany). Similarly, the fluorescence signal from the P35S::GFP construct in magnetofected pollen was examined using a laser scanning confocal microscope [25]. Fluorescence images were blindly scored for GFP expression in pollen grains and pollen tubes.

### 4.5. Generation, Screening, and Analyses of Transgenic Plants

The nanomagnetic beads transformation method comprised mild conditions and had little effect on pollen viability, thus the transformed pollen should be suitable for cross-pollination. The transformed pollen was germinated in vitro and a pollen grain was regarded as germinated if its tube’s length exceeded the diameter of the pollen grain. Owing to the poor pollination utility of wet pollen, it was necessary to collect and dry the transformed pollen before cross-pollination. A 500-mesh nylon cloth was used to filter and dry the pollen. The pollen viability before and after drying was assessed under the same germination conditions as used previously. The average percentage pollen germination in three microscopic fields was calculated for the transformed pollen and the nontransformed pollen of the control.

*Lilium regale* flowers were manually emasculated before anther dehiscence and the stigma was protected with silver paper. Once mucus was secreted from the stigma (normally 2–3 days after blooming, which is the optimal time for pollination), magnetofected pollen grains were manually placed on the stigmas to generate transgenic seeds. The seeds were sown and grown into seedlings in a peat moss substrate under a greenhouse. The temperature ranged from 20 to 25 °C, with an average light intensity of 91.37 μmol/m^2^/s. The seedlings raised from the transformed seeds were analyzed by PCR and RT-PCR (for details, see Table 10 and Table 11).

### 4.6. Statistical Analysis

Corresponding brightfield images were used to obtain total pollen counts. Transformation efficiencies were calculated as the total number of transformation events divided by the total number of pollen grains in which transformation was attempted in each treatment. Duncan’s multiple range test was used to calculate *p*-values relative to the positive control for each individual trial. The experiment was conducted three times.

The SPSS 18.0 software was used for the statistical analyses. One-way analysis of variance (ANOVA) was used to evaluate whether differences in pollen characteristics from different provenances were significant. For cases in which ANOVA detected a significant difference, a least significant difference test was performed to compare the individual means (Table 2).

### 4.7. SEM Analysis

Morphological observations on lily pollen grains were conducted using SEM. The pollen grains were directly attached to double-sided adhesive tape and examined under a stereomicroscope to locate the pollen. The sample was then taped to the object stage. Following sputter coating with gold, sample observation and image acquisition were conducted using a Hitachi S-3400 scanning electron microscope (Tokyo, Japan) following a previously described method [37]. All microscopy procedures were performed at the Biotechnology Centre, Beijing Forestry University, China.

The pollen biometric data were measured using Image-Pro Plus 6.0 (Media Cybernetics, Silver Spring, MD, USA). For each sample, 10 fully developed pollen grains (including five *L. brownii* pollen grains from Shennongjia, Hubei Province, China) were measured. Indicators measured were P, E, P/E ratio, P·E, LD, and muri width. The description of the pollen morphology was based on the shape and sculpturing classifications of Baranova [22]. The data are presented as the mean and standard deviation for each indicator.

## 5. Conclusions

In summary, we report a modified method for transient transformation via pollen magnetofection for *Lilium* germplasm. Using the modified protocol, transformed pollen and transgenic seedlings were successfully obtained and verified. The results suggested that the pollen magnetofection transformation method can be applied generally to members of the genus *Lilium* but might exhibit a degree of species or cultivar specificity. Importantly, the pollen E values showed a positive correlation, P/E values a negative correlation, and P values showed no significant correlation with transformation efficiency.

## Figures and Tables

**Table 1:** *Lilium regale* pollen germination percentage in four liquid media.

Medium Formula (1 L)	Pollen Germination Rate (%)
H_3_BO_4_ 30.5 mg + CaCl_2_ 30.5 mg + Sucrose 100 g	46.40 ± 1.28 ^a^
H_3_BO_4_ 61.5 mg + CaCl_2_ 61.5 mg + Sucrose 100 g	40.33 ± 2.51 ^b^
H_3_BO_4_ 61.5 mg + CaCl_2_ 91.5 mg + Sucrose 100 g	85.73 ± 0.84 ^c^
H_3_BO_4_ 30.5 mg + CaCl_2_ 91.5 mg + Sucrose 100 g	59.77 ± 3.80 ^c^

Note: Superscripted lowercase letters indicate statistically significant differences between different stages (*p* < 0.05).

**Table 2:** Percentage staining for GUS activity of *Lilium regale* pollen under different transformation conditions.

Ratio of MNPs and Plasmid DNA	Transformation Time (h)		
	0.5	1.0	1.5
1:4	85.50 ± 0.07 ^a^	82.10 ± 0.12 ^a^	83.47 ± 0.18 ^a^
1:1	74.50 ± 0.74 ^c^	75.50 ± 0.72 ^c^	73.77 ± 0.95 ^c^
3:2	81.50 ± 1.77 ^b^	79.81 ± 0.85 ^b^	81.0 ± 0.32 ^a^

Note: MNPs, magnetically driven plasmids. Superscripted lowercase letters indicate statistically significant differences between different stages (*p* < 0.05).

**Table 3:** Percentage germination of *Lilium regale* pollen under different treatments.

Treatments	Pollen Germination Rate (%)
Untreated pollen	62.65 ± 0.97
Magnetofected pollen	61.62 ± 0.39
Magnetofected pollen after dry treatment	60.77 ± 0.22

**Table 4:** Transformation efficiency for the PBI121 plasmid DNA in *Lilium regale* pollen.

	No Magnetofection, No DNA	Magnetofection, No DNA	Magnetofection, with PBI121 Plasmid DNA
Total transformants (pollen grains and pollen tubes)	3	2	726
Total pollen in imaged fields	748	632	822
Transformation efficiency	0.40%	0.32%	88.32%
*p*-value		0.000	0.002

**Table 5:** Transformation efficiency for the pYBA1132 plasmid DNA in *Lilium regale* pollen.

	No Magnetofection, No DNA	Magnetofection, No DNA	Magnetofection, with pYBA1132 Plasmid DNA
Total transformants (pollen tubes)	0	0	264
Total pollen in imaged fields	332	319	379
Transformation efficiency	0.00%	0.00%	69.66%
*p*-value		0.000	0.003

**Table 6:** Transformation efficiency for the PBI121 plasmid DNA in *Lilium regale* seedlings.

	Control	GUS Staining Results
Total transformants (seedlings)	0	151
Total seedlings	100	268
Transformation efficiency	0%	56.34%
*p*-value		0.000

**Table 7:** Morphological characteristics of lily pollen grains.

No.	Samples	P (μm)	E (μm)	P/E	P·E (μm^2^)	Muri Width (μm)	Sculpture	LD
1	*Lilium leichtlinii* var. *maximowiczii*	87.10	37.37	2.34	3250.49	1.74	Nodular	uniform
2	*Lilium davidii* var. *willmottiae*	94.47	32.60	3.09	3112.71	2.02	Disc bead	uniform
3	*Lilium leucanthum*	100.00	39.77	2.53	3992.85	2.31	Disc bead	nonuniform
4	*Lilium henryi*	87.87	35.37	2.50	3121.79	1.93	Disc bead	uniform
5	‘Bright Tower’	113.10	53.67	2.11	6097.52	1.48	Disc bead	nonuniform
6	‘Yellow Diamond’	101.57	49.03	2.09	4994.39	2.10	Disc bead	uniform
7	‘Pink Flavor’	99.00	36.60	2.71	3626.73	1.97	Disc bead	nonuniform
8	‘Bonfire’	133.83	58.73	2.28	7877.28	2.08	Disc bead	uniform
9	‘Beverly’s Dream’	104.13	45.30	2.30	4722.80	1.43	Disc bead	uniform
10	‘Cancun’	85.57	36.60	2.34	3134.58	1.17	Disc bead	uniform
11	‘Leichtlinii’	67.77	39.33	1.75	2673.94	1.83	Nodular	uniform
12	‘Bellring’	112.07	55.03	2.16	6155.96	3.30	Disc bead	nonuniform
13	‘Millesimo’	108.90	49.03	2.22	5345.02	1.93	Disc bead	uniform
14	‘Sunderland’	122.57	70.43	1.74	8643.40	2.05	Disc bead	nonuniform
15	‘Mister Job’	122.50	51.00	2.42	6272.38	2.05	Nodular	uniform
16	‘Sweet Surrender’	94.37	34.40	2.76	3255.88	1.75	Disc bead	nonuniform
17	‘Teresina’	97.17	40.20	2.43	3920.88	1.73	Nodular	nonuniform
18	‘Regale’	106.33	45.40	2.34	4831.05	2.40	Disc bead	nonuniform
19	‘Tresor’	115.87	39.40	2.98	4626.59	2.34	Nodular	uniform
20	‘Fusion’	92.90	38.40	2.42	3572.34	1.96	Nodular	nonuniform

P: polar axis; E: equatorial axis; MW: muri width; LD: lumina distribution.

**Table 8:** Transformation efficiency of pollen from 20 lily taxa and hybrids.

No.	Name	Magnetofection Transformation Efficiency (%)	Control Transformation Efficiency (%)
1	*Lilium leichtlinii* var. *maximowiczii*	2.41 ± 4.09 ^b^	0.00 ± 0.00 ^a^
2	*Lilium davidii* var. *willmottiae*	0.00 ± 0.00 ^a^	0.00 ± 0.00 ^a^
3	*Lilium leucanthum*	54.47 ± 7.87 ^bc^	5.00 ± 2.11 ^bc^
4	*Lilium henryi*	9.23 ± 4.87 ^b^	0.00 ± 0.00 ^a^
5	‘Bright Tower’	0.00 ± 0.00 ^a^	0.00 ± 0.00 ^a^
6	‘Yellow Diamond’	0.50 ± 0.87 ^a^	0.00 ± 0.00 ^a^
7	‘Pink Flavor’	0.00 ± 0.00 ^a^	0.04 ± 0.08 ^a^
8	‘Bonfire’	0.00 ± 0.00 ^a^	0.00 ± 0.00 ^a^
9	‘Beverly’s Dream’	19.80 ± 4.85 ^b^	1.27 ± 1.25 ^ab^
10	‘Cancun’	23.33 ± 8.75 ^bc^	0.00 ± 0.00 ^a^
11	‘Leichtlinii’	0.00 ± 0.00 ^a^	0.00 ± 0.00 ^a^
12	‘Bellring’	4.53 ± 3.06 ^b^	0.00 ± 0.00 ^a^
13	‘Millesimo’	0.00 ± 0.00 ^a^	0.00 ± 0.00 ^a^
14	‘Sunderland’	0.40 ± 0.69 ^a^	0.00 ± 0.00 ^a^
15	‘Mister Job’	1.00 ± 1.73 ^a^	0.00 ± 0.00 ^a^
16	‘Sweet Surrender’	85.80 ± 5.85 ^b^	6.67 ± 3.61 ^c^
17	‘Teresina’	46.22 ± 19.23 ^c^	0.00 ± 0.00 ^a^
18	‘Regale’	68.53 ± 12.36 ^bc^	3.13 ± 1.99 ^c^
19	‘Tresor’	55.90 ± 20.34 ^c^	0.00 ± 0.00 ^a^
20	‘Fusion’	0.00 ± 0.00 ^a^	0.00 ± 0.00 ^a^

Note: Superscripted lowercase letters indicate statistically significant differences between different stages (*p* < 0.05).

**Table 9:** Pearson correlation analysis of transformation efficiency with pollen grain size and shape indicators from 20 lily taxa and hybrids.

	P (μm)	E (μm)	P/E	P·E (μm)^2^
Transformation efficiency (%)	−0.232	−0.362 **	0.321 **	−0.307 *

Note: E, pollen equatorial diameter; P, polar axis length. * *p* < 0.05, ** *p* < 0.01.

**Table 10:** GUS gene validation primers for transformants.

No.	Primer Name	Primer Sequence (5′-3′)	Tm (°C)
1	*35sgF*	ACCTCCTCGGATTCCATTG	53.5
2	*35sgR*	AACATACGGCGTGACATCG	57.5

**Table 11:** The PCR verification reaction system.

No.	Reagent Name	Reagent Dosage (μL)
1	rTaq	0.2
2	10×buffer	2.5
3	dNTP	2
4	Templates	1
5	35sgF	1
6	35sgR	1

## Data Availability

No new data were created or analyzed in this study. Data sharing is not applicable to this article.

## Supplementary Materials

The following supporting information can be downloaded at: https://www.mdpi.com/article/10.3390/ijms242015304/s1.
