# Supplementary material for: A Modified Method for Transient Transformation via Pollen Magnetofection in Lilium Germplasm
Source: Int J Mol Sci. 2023 Oct 18;24(20):15304. doi: 10.3390/ijms242015304 (PMC10607007; doi:10.3390/ijms242015304)
Supplement: Supplementary file 1 [file ijms-24-15304-s001.zip › ijms-2544273-supplementary.pdf]

**Supplementary Materials:**

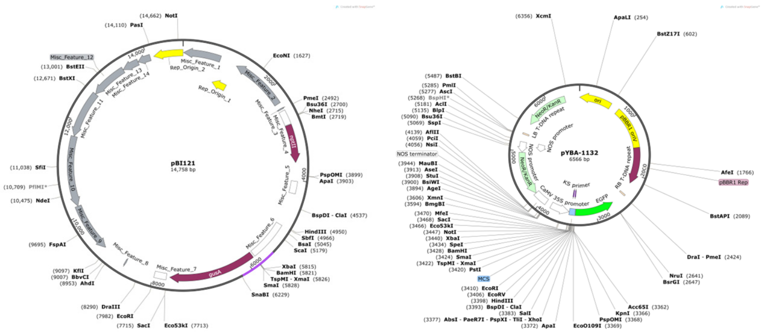


**Figure S1.** The vector map of pBI 121 and pYBA1132.

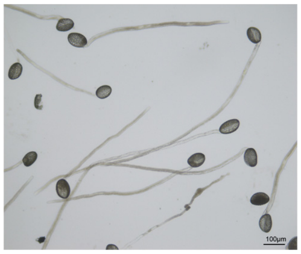

(**a**)

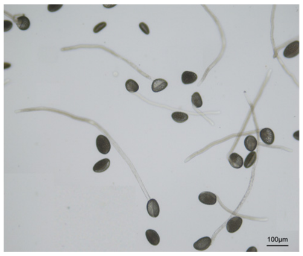

(**b**)

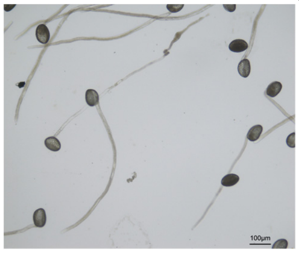

(**c**)

**Figure S2.** Germination status of *Lilium regale* pollen under different treatments. (**a**) Untreated pollen; (**b**) Magnetofected pollen; (**c**) Magnetofected pollen after dry treatment.

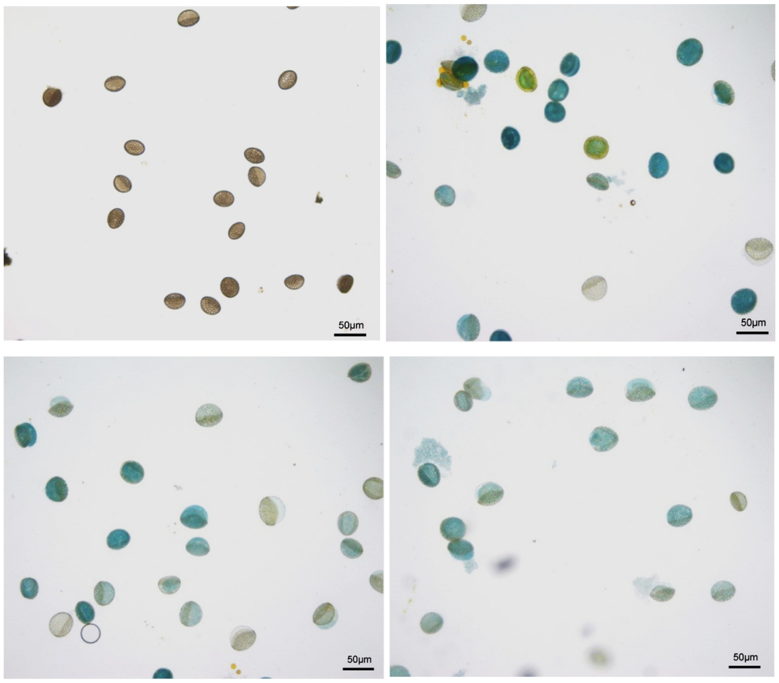


**Figure S3.** The GUS activity tested under different conditions.

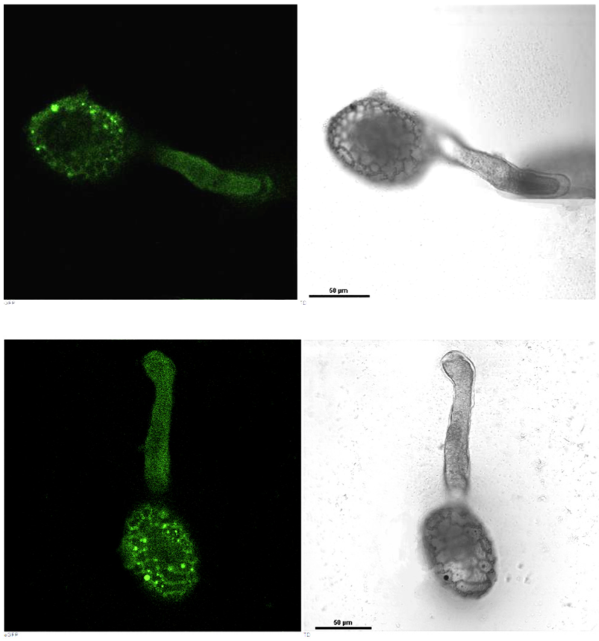

**Figure S4.** More images of the GFP activity observed in pollen germination tube.

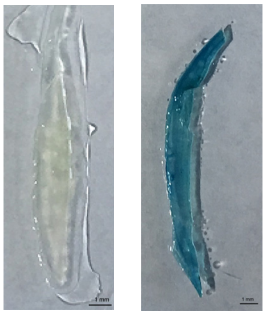

(**a**) (**b**)

**Figure S5.** Detection of β-glucuronidase activity in seedling progeny from self-pollination of L. regale using magnetofected pollen. (**a**) Control seedling leaf; (**b**) transformed seedling leaf.
